# Stereosonic vision: Exploring visual-to-auditory sensory substitution mappings in an immersive virtual reality navigation paradigm

**DOI:** 10.1371/journal.pone.0199389

**Published:** 2018-07-05

**Authors:** Daniela Massiceti, Stephen Lloyd Hicks, Joram Jacob van Rheede

**Affiliations:** 1 Department of Engineering Science, University of Oxford, Oxford, United Kingdom; 2 Nuffield Department of Clinical Neurosciences, University of Oxford, Oxford, United Kingdom; 3 Department of Physiology, Anatomy and Genetics, University of Oxford, Oxford, United Kingdom; Pennsylvania State University, UNITED STATES

## Abstract

Sighted people predominantly use vision to navigate spaces, and sight loss has negative consequences for independent navigation and mobility. The recent proliferation of devices that can extract 3D spatial information from visual scenes opens up the possibility of using such mobility-relevant information to assist blind and visually impaired people by presenting this information through modalities other than vision. In this work, we present two new methods for encoding visual scenes using spatial audio: simulated echolocation and distance-dependent hum volume modulation. We implemented both methods in a virtual reality (VR) environment and tested them using a 3D motion-tracking device. This allowed participants to physically walk through virtual mobility scenarios, generating data on real locomotion behaviour. Blindfolded sighted participants completed two tasks: maze navigation and obstacle avoidance. Results were measured against a visual baseline in which participants performed the same two tasks without blindfolds. Task completion time, speed and number of collisions were used as indicators of successful navigation, with additional metrics exploring detailed dynamics of performance. In both tasks, participants were able to navigate using only audio information after minimal instruction. While participants were 65% slower using audio compared to the visual baseline, they reduced their audio navigation time by an average 21% over just 6 trials. Hum volume modulation proved over 20% faster than simulated echolocation in both mobility scenarios, and participants also showed the greatest improvement with this sonification method. Nevertheless, we do speculate that simulated echolocation remains worth exploring as it provides more spatial detail and could therefore be more useful in more complex environments. The fact that participants were intuitively able to successfully navigate space with two new visual-to-audio mappings for conveying spatial information motivates the further exploration of these and other mappings with the goal of assisting blind and visually impaired individuals with independent mobility.

## 1 Introduction

Globally more than 250 million people are visually impaired, with over 35 million of this group classified as blind [1, 2]. While certain causes of visual impairment can be prevented or treated, a large proportion of sight loss remains without a cure [3]. New treatment approaches such as retinal prosthetics, optogenetics, and gene therapy offer hope for the future, but at present are at a research or early implementation stage and await evidence of real-life benefit to patients [4].

Vision loss affects the ability to independently carry out activities of daily living [5–8], in part due to its negative impact on mobility and navigation [9–15]. While blind or visually impaired individuals are often able to learn to successfully navigate without vision through *orientation and mobility* training [16], they face significant challenges not faced by the sighted population [17–20]. Non-sighted navigation requires more planning and cognitive resources [19–22], and blind and visually impaired individuals are at an increased risk of mobility-related accidents, injuries, and falls [23–27]. Even when walking around a familiar environment, the variable presence of obstacles, changes in walking surface or drop-offs can be a significant perceived mobility hazard [28], and even very experienced non-sighted navigators still regularly veer off their intended course [29]. It also remains the case that human living spaces are usually designed with sighted navigation in mind [19].

While academic debate exists around the representation of spatial information in blind individuals, in particular people who are congenitally blind, it is clear that the cognitive ability for representing spatial information is not the main limiting factor in navigation and mobility [20, 30]. Rather, the limitation lies in the rate at which non-sighted navigators are able to *acquire* spatial information about their current environment, whether that is in order to build an initial cognitive map of the space, or to update their current position in a cognitive map from memory and scan for the presence of any obstacles to safe mobility [20]. While vision is uniquely well-placed to rapidly provide mobility-relevant spatial information [31], it is not the only source of such information. Already, many blind individuals will spontaneously learn to use non-visual cues to their advantage in sensing obstacles in their environment [32, 33].

Sensory Substitution Devices (SSDs) go one step further, converting information normally acquired through one sensory modality into a representation that is compatible with another intact sensory modality, aiming to exploit the increasingly well-understood cross-modal capacities of the brain [34, 35]. Approaches to substituting information received through vision naturally focus on the other spatially informative senses, namely hearing and touch [36]. The first SSDs were pioneered by Bach-y-Rita in the 1960s with his development of the Tactile Vision Sensory Substitution (TVSS) device [37]: subjects received vibrating patterns via an array of pins mounted on their backs and were able to differentiate between oriented parallel lines, simple geometric shapes and capital block letters. Extending this initial work, a host of studies have investigated “seeing” with vibro-tactile and electro-tactile stimulation applied to a number of body surfaces [38–41]. Other SSDs have attempted to present an auditory representation of the visual scene. The *vOICe*, perhaps the most widely investigated vision-to-audio SSD, scans the visual environment from left to right, and converts the 2D grayscale image into a frequency spectrum or “soundscape” [42]. These efforts and others like EyeMusic [43] have largely focused on converting a 2D camera image into a corresponding sound or set of sounds for the purpose of identifying elements of the visual scene.

Mobility, however, is a 3D task, and requires access to information about the distance of objects in the visual scene and their radial position. An effective SSD for navigation and mobility will therefore need to provide such information as explicitly as possible. This moves away from the concept of an SSD as a generic replacement for vision, and towards seeing it as a mobility tool aimed at providing the user with an improved spatial awareness of their surroundings. This task-specific approach has the additional advantage of reducing the bandwidth of the information needed to be encoded cross-modally—an important consideration [36, 44]. Several SSDs have been developed specifically with this in mind (for detailed surveys of such devices, see [36, 44, 45]). Work in this field has initially been led by Leslie Kay with the initial Kay Sonic Torch [46] followed by several others [47–51]. While the first approaches only provided users with a ‘virtually extended’ cane that reported the distances of objects further afield in their path, the more ambitious devices use a frequency-modulated ultrasound sweep of the environment to provide a true ‘soundscape’.

Despite the active research in this area and some encouraging results in academic settings, the uptake of SSDs for mobility by the blind and visually impaired community has been low [44, 45, 52]. Giudice and Legge [44] identify four factors where SSDs can fail: 1) the sensory translation rules—how effectively does the sensory translation convey the relevant information in another modality?, 2) selection of the information to be transcoded between senses—is the information that is being translated actually the most adequate for carrying out the task at hand?, 3) is the device practical to use?, and finally, 4) does the device have an acceptable form factor? We believe that the availability of new, affordable, and portable devices that can sense and reconstruct the 3D environment and extract semantic information from it (discussed below) allows for novel approaches to sensory translation rules and the selection of task-relevant information that could be implemented on devices that are both practical and unobtrusive. In this work, we focus on sensory translation rules and information selection.

For a dynamic and time-constrained task such as navigation, there is a great need for sensory translation rules to be intuitive. In this work, we have therefore focused on visuospatial-to-audio sensory substitution as this has the advantage that we already use auditory information to inform our representations of near space: we are able to rapidly and accurately localise sound sources based on level differences, temporal delays, and their spectral envelope [53], and use this on a daily basis to direct our attention, head and gaze towards sound sources. Therefore, inferring spatial information from such cues should come naturally. In contrast, an array of stimulators on a patch of skin may have a natural correspondence with, for instance, the type of 2D image produced by a camera, but there is no such natural correspondence between a 2D patch of the skin and near space (beyond the immediate body surface).

This leaves the question of how to best convey such visuospatial information to the wearer via audio—a process we will refer to as ‘sonification’. Here, research thus far has taken inspiration from blind individuals such as Daniel Kish [54] who have learned to use echolocation for navigation and mobility (for recent reviews on the psychophysics of human echolocation see [55, 56]). Echolocation refers to the general process of using sound reflections to infer the spatial properties of the environment. Typically, human echolocators will use a sharp, self-generated sound such as a mouth click to sample their surroundings, and use properties of the sound reflections such as their volume, delay, spectral properties, and stereo components (left versus right ear) to infer the spatial structure of the environment [55, 57]. It is a skill that takes a long time to develop and is best learnt at a young age [58], but the signal provided contains the right sort of spatial information needed for navigation. Previous SSDs have tried to make echolocation less disruptive to the auditory environment by emitting ultrasonic audio pulses, and making it easier to learn by playing the user a slowed-down recording of the echoes in the audible frequency range [59–63].

These SSDs work by presenting a spatially informative modified audio signal to the user, but they have no access to a 3D model of the user’s surroundings. This limitation can be addressed, however, by the recent proliferation of portable devices that can rapidly scan and reconstruct 3D environments (e.g. through stereoscopic depth or using active projection of infrared features such as in the XBox Kinect) [64–67]. Such explicit access to information about the spatial structure and semantic context of the environment opens up the possibility of conveying this information via non-visual sensory modalities in a manner that is independent of how such information was acquired. It follows, therefore, to consider whether there are sonification strategies that would be easier to learn or more informative than those directly derived from the strategy used to sample the environment with human-generated echolocation. Additionally, having access to a 3D reconstruction of the environment means it is now possible for devices to represent objects that are currently outside the ‘field of view’ of the device, enabling the creation of persistent audio beacons representing targets or obstacles around the wearer.

In this work, we have explored two novel, relatively simple and sparse spatial audio representations of 3D environments: 1) simulated echolocation with discrete ‘sound particles’ and 2) distance-dependent hum volume modulation of beacon sounds attached to objects. Our simulated echolocation acknowledges the previous work that has been carried out in this area, but aims to present a less complex sample of the environment by having the user virtually emit a fixed number of particles in a 90x90 degree field of view determined by their head direction. The emitted particles ‘pop’ (i.e. are sonified) as they make contact with virtual obstacles in the user’s nearby vicinity, and in this way are analogous to the echoes of sound that would bounce of real-world objects in traditional echolocation. Through the particles’ time-of-flight delays (captured as the temporal delay between an initiating click sound and a particle’s pop as it bounces off an obstructing object) and the volume and stereo components of the pop sound itself, 3D spatial information about the environment is conveyed to the user. The object-centric distance-dependent hum volume modulation, on the other hand, departs from the principles of echolocation, and instead transforms features of the environment itself—in our case, virtual obstacles and walls—into sources of sound. Each type of object is assigned a humming sound of characteristic frequency, and the volume of the hum is modulated by the user’s distance to the object, with a higher volume indicating a shorter distance.

To test these mappings for the task of spatial navigation, we used a novel auditory virtual reality (VR) paradigm. The potential of VR for rapid prototyping and testing of audio-based navigation has been recognised previously [68–71]. It allows researchers to generate any number of arbitrary and randomised environments for participants to navigate, and enables rapid and precise extraction of the dynamics of navigation and mobility behaviour [68]. Moreover, using virtual obstacles avoids exposing participants to real mobility hazards. Some have focused on understanding the neural mechanisms of navigation and developed paradigms that translate well to neuroimaging settings [69], capitalising on the ability of the subject to remain still while navigating in virtual space. This, however, does not take into account the important contributions of proprioceptive inputs to navigation [72, 73]. For the development of a navigational aid that ultimately aims to have a real-world implementation, it is important to incorporate such proprioceptive cues, especially as it is to be expected that the importance of these inputs becomes magnified in the absence of vision.

Until recently, accurate locational tracking in VR required a substantial amount of dedicated infrastructure (e.g. as in the SWAN system used by Walker and colleagues [68, 74–76]). Here, however, we present a portable implementation of an immersive VR with the ability to accurately track the movements of users wearing a cord-free VR headset. More specifically, we employed a tablet computer called the *Google Tango* which accurately tracks its 3D position and rotation, and this tablet was worn as a head-mounted device by blindfolded sighted participants. We created two types of VR environment, viewed live through the VR tablet headset, which participants could navigate through by physically walking, ensuring realistic proprioceptive mobility feedback. In each of the two environments, the blindfolded participants were tasked with navigating to an end point (unknown a priori), but crucially were presented *only* the audio cues for the sonification mapping being tested via stereo headphones. For each of the two environments, navigation using the vision-to-auditory mappings was compared against a visual baseline condition: participants, without blindfolds, performed the navigation tasks under similar settings, with the end points randomised and thereby still unknown a priori. Central to our quantitative assessment of participants’ navigational efficiency was the 3D tracking capability of the *Google Tango* tablet. The device captured the real-time 3D dynamics of participants’ walking behaviour in the VR environments, thus enabling us to develop mobility-relevant metrics and provide an in-depth analysis of participants’ movements in both auditory conditions and the visual baseline condition.

In summary, our three main research aims with this work were:

- To develop new visual-to-audio mappings that simultaneously provide information about obstacle distance and radial position relative to the wearer;
- To develop a locomotion-controlled, flexible navigation paradigm to test whether these new mappings are in principle sufficient for navigating an environment using sound alone;
- To investigate whether there are differences in participants’ navigation performance, strategies and speed of learning between the two sonification approaches.

Ultimately, the aim of exploring these new sonification strategies is to establish their suitability for application either using stand-alone devices or in depth-based electronic travel aids [77, 78] to assist independent navigation and mobility in blind and visually impaired individuals.

## 2 Methodology

### 2.1 Participants

18 participants were recruited locally in Oxford, United Kingdom. Participants were healthy volunteers with full sight and full stereo hearing. The choice to test a fully-sighted participant group had a twofold motivation: firstly, the test group was able to provide a visual control condition in our experiments by performing the navigation task with access to visual information in each virtual environment. Secondly, the group was seen as a necessary first step given that this is a proof-of-principle study exploring novel sonification methods for 3D spatial representation and navigation within a new experimental paradigm.

The mean age of participants was 28.78 ± 8.00 with a male/female distribution of 11/7. All participants had normal or corrected-to-normal vision. Due to the physical limitations imposed by the testing equipment, it was necessary that participants with corrected vision were able to wear contact lenses for the testing rather than glasses. Participants were rated on the amount of experience they had had with the developed SSD prior to the testing and their experience with first-person-controller computer games and virtual-reality devices (Table 1).

**Table 1:** Participant demographics. Naivety rated on 1-5 scale of experience: 1 (> 9 hours), 2 (> 6 hours), 3 (> 3 hours), 4 (> 0 hours), 5 (= 0 hours). Virtual-reality/gaming frequency rated 1-5: 1 (no experience), 2 (rarely), 3 (several times a year), 4 (monthly), 5 (regularly).

	Sex	Naivety	Age	Corrected vision	Vision without glasses/ contacts	Virtual reality/gaming experience
1	M	4	40	Y	Y	5
2	M	5	28	N	-	2
3	F	3	23	Y	Y	2
4	M	5	24	N	-	4
5	F	5	24	N	-	1
6	F	5	27	Y	Y	2
7	M	5	19	Y	Y	3
8	M	5	32	Y	Y	2
9	M	5	28	N	-	5
10	F	5	30	Y	Y	1
11	F	5	24	N	-	2
12	F	5	26	N	-	2
13	F	5	31	Y	Y	2
14	M	5	25	Y	Y	3
15	M	5	29	N	-	2
16	M	5	23	N	-	3
17	M	5	30	N	-	2
18	M	5	55	Y	Y	1

On the day of testing, participants were verbally instructed on the types of environments that they would be required to navigate (Section 2.2), the two sonification methods to be tested (Section 2.3), the equipment to be used (Section 2.4) as well as the experimental protocol (Section 2.5). Testing was conducted over two 1.5 hour sessions per participant. A brief re-instruction was conducted at the start of the second session. Verbal feedback was collected during and after each session. In addition to this, a voluntary and anonymous follow-up survey was sent to participants after their completion of both sessions.

**Ethics** This work received ethical clearance through the University of Oxford Central University Research Ethics Committee. All individuals involved in the study provided written informed consent to publish these case details.

### 2.2 Virtual reality environments

Participants were tasked with spatially navigating to randomised end points in VR environments, using each of the developed visual-to-audio mappings (sonification conditions) or using visual information (visual-only baseline condition). Two types of VR environments were constructed: a maze and an obstacle corridor. Our motivation for using virtual environments to test our methods was two-fold:

1. Virtual environments offer the ability to build randomised environments and obstacles on the fly which is an advantage over real-world testing.
2. Using a virtual environment bypasses the problem of detecting the 3D structure of the environment, allowing us to focus on the methods of conveying such spatial information to our participants.

**Fig 1:**
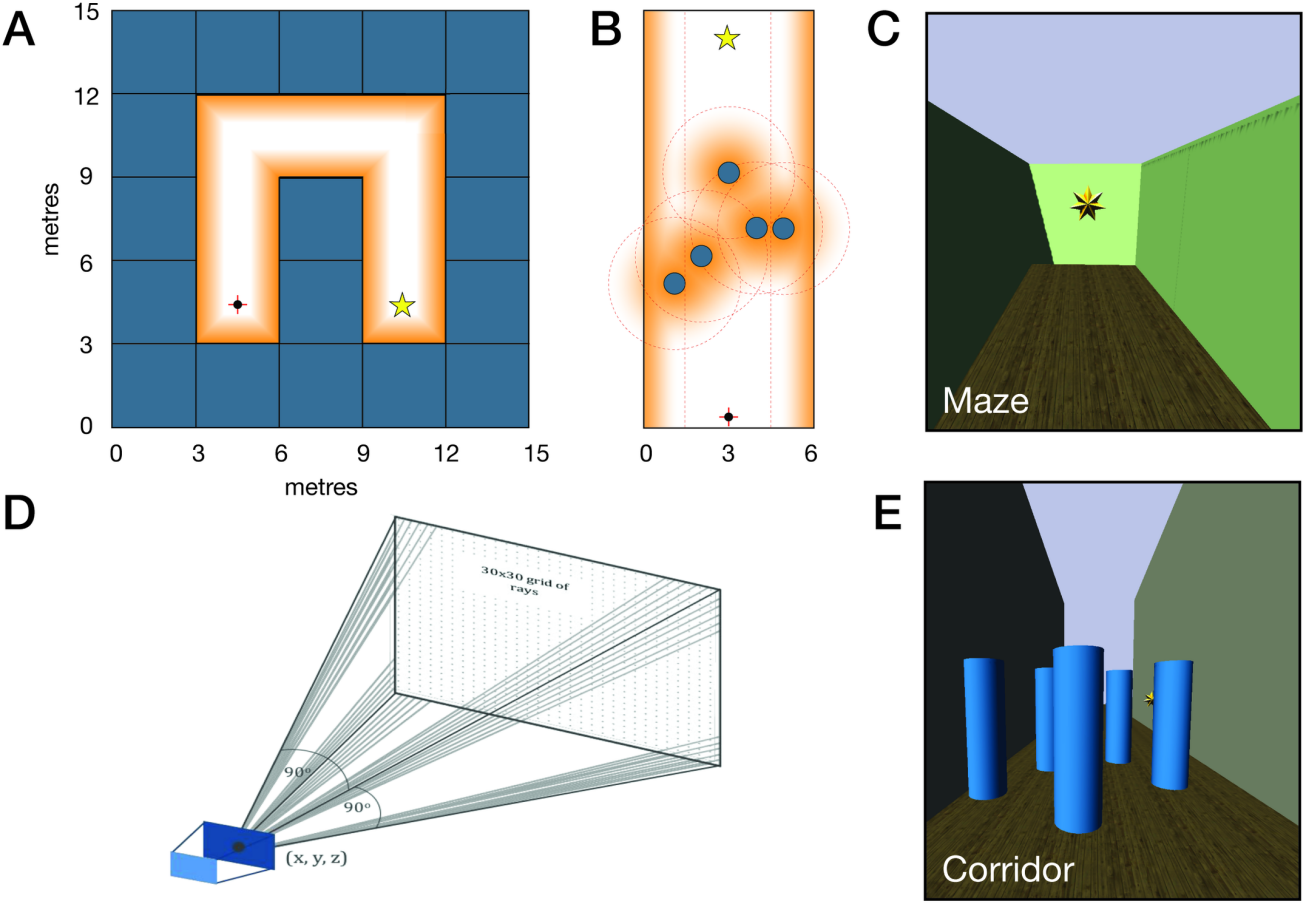
Virtual reality visualisation and implementation of simulated echolocation and distance-dependent hum volume modulation (humming). (A) Example of a randomly-generated maze with a single path to goal of 7 cube steps. Orange glow indicates humming boundary in the humming sonification. Golden star indicates goal. Red cross + black dot indicate starting position. (B) Example of a corridor with randomly positioned obstacles. Orange glows and red dotted lines indicate humming boundaries in the humming sonification. Golden star indicates goal. Red cross + black dot indicate starting position. (C) Virtual reality visualisation of the maze. (D) Implementation of simulated echolocation. A pool of 900 particles is projected along a 30 × 30 grid array of rays from the (x, y, z) position of the *Tango*. (E) Virtual reality visualisation of the obstacle corridor.

**Maze.** The maze environments were generated within a 5 × 7 grid of virtual cubes, each cube 3 × 3 × 3*m* in size making a real-world sized arena of 15 × 21 × 3*m*. Each maze was constructed such that there existed only one constant-length (7 cubes) path to a goal which was a golden star (Fig 1A and 1C). For each maze trial, the path was randomly selected from a pool of 20 pre-generated maze paths saved on the *Tango* tablet (see Section 2.4). Upon selection, the participant was placed at the starting point of the selected virtual maze.

**Obstacle corridor.** The maze environment simplifies the task of navigation to making a series of right-angled turns. To mimic a scenario closer to the real-world challenge of detecting and avoiding randomly-placed obstacles, based on the work of [78], we constructed obstacle corridor environments. These virtual environments were laid out in a 6m-wide corridor bounded by a left and right wall with the length of the corridor segmented as follows: an initial 3m of empty space, a 7m segment of obstacles, a second 3m of empty space, and finally the goal, again a golden star. For each trial, the obstacle segment was populated by 5 randomly-positioned columnar objects of 0.8m diameter and 1m height. Additionally, for each trial, the star was placed at 13m from the starting line at a random point along the corridor’s 6m width (Fig 1B and 1E).

The placement of obstacles was randomised such that there were always multiple possible paths through the obstacles to the goal, and these paths were sufficiently diverse and also non-trivial (for example, participants could not simply navigate around the edges of the obstacle segment where they would encounter no obstacles). As with the mazes, for each trial, the obstacle corridor arrangement was randomly selected from a pool of 20 pre-generated arrangements saved on the *Tango*. Upon selection, the corridor was dynamically constructed and the participant placed at the starting point in virtual space.

### 2.3 Stereosonic vision

Our aim was to convert the 3D structure of the virtual visual environment into a stereo soundscape providing spatial information to our participants for the task of navigation. We call this stereosonic vision. This section presents the two visual-to-audio mappings, or sonification methods, we implemented and explored in the VR environments: the first, a simulated echolocation and the second, a distance-dependent hum volume modulation of hums attached to objects. Resulting sounds were presented on stereo headphones worn by the participant.

#### 2.3.1 Simulated echolocation

There is a large body of work supporting echolocation as a useful mobility technique for blind people [55, 57, 79]. Using self-generated sounds such as mouth clicks, echolocators use sonic reflections to infer the spatial properties of their surroundings. Learning to navigate using echolocation in the real world, however, is difficult and takes much training [55, 56, 58]. In this work, we simulated echolocation in the two VR environments. This alleviated the real-world difficulties of echolocation since we were able to slow down the speed of echolocation feedback, and also select the types of echo sounds. Our motivation was to make simulated echolocation easier to interpret and acoustically more pleasant than real world echolocation. The following describes our VR implementation of simulated echolocation, which from here on we will refer to as simply ‘echolocation’.

**Fig 2:**
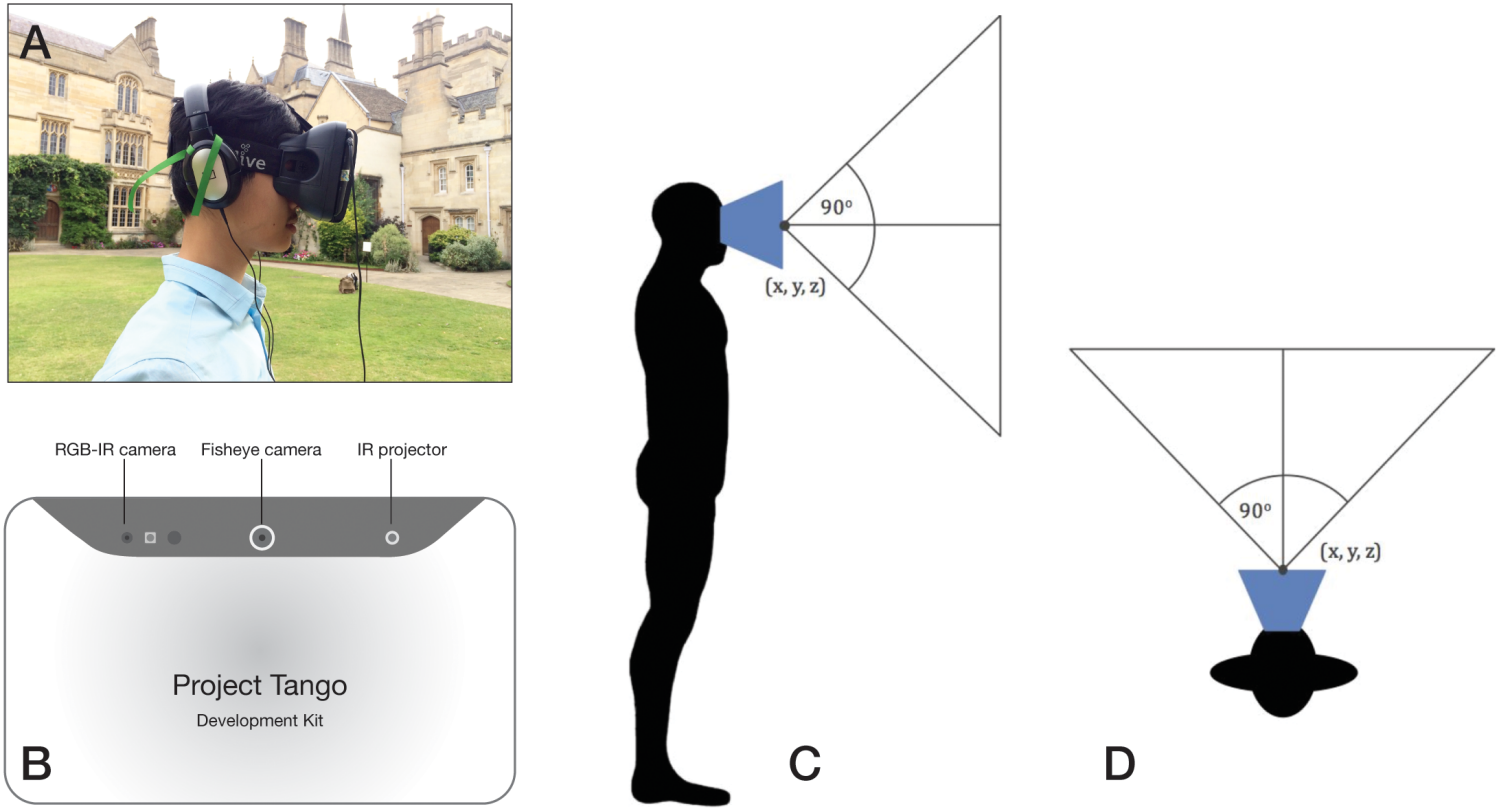
Hardware set-up with the *Project Tango* and the *Durovis Dive 7* head mount. (A) Participant wearing the *Tango* mounted in the *Durovis Dive 7* headset with a pair of stereo headphones. (B) Rear-facing camera hardware of *Project Tango* tablet. (C & D) 90° field of view vertically and horizontally from the position of the *Tango*.

Much like echolocation in the real world, we implemented simulated echolocation in the virtual world such that it was dependent on the user’s body and head direction. The *Tango* emitted a virtual “chirp” in the form of a sharp click (audible via the user’s headphones) at a rate of 0.57*Hz*, or every 1.75 seconds. With each click, a pool of 900 virtual particles was projected radially outwards from the (x, y, z) position of the *Tango* (where x and y were the lateral coordinates of the *Tango* in space, and the z coordinate was its height above the ground). Each of the 900 particles was projected along its own radial line, following a 30 × 30 grid arrangement (Fig 1D). The radial projection of the particles had a field of view (FOV) of 90° vertically and horizontally (Fig 2C and 2D) with the rays spaced 3° apart in the horizontal and vertical direction, respectively. As the *Tango* was moved through space, the pool of particles was recast from the *Tango*’s updated (x, y, z) position. Since the *Tango* was head-mounted on the participant (see Section 2.4 and Fig 2A), a head/body rotation caused a corresponding *Tango* rotation. In this way, the virtual particle projection was always outwards and forwards relative to the participant’s head direction.

Following a click, each particle within the pool was projected along its ray but only generated a sound, again audible via the user’s headphones, if it ‘bounced’ off a virtual object in the VR environment. This was intended to be analogous to the echoes of sounds that would be reflected off real-world objects in traditional echolocation. In terms of implementation in our sound engine, this simply meant that a particle was ‘silent’ when travelling along its ray trajectory and emitted its sound when a virtual object in the virtual world obstructed that trajectory. This was thresholded to virtual objects within a 3m radius of the *Tango*’s (x, y, z) position. The sound of a particle bouncing off an object was sonified as a “pop”, intended to be harmonious and easy on the ear. The pairing of clicks and pops aimed to convey three pieces of information about the 3D spatial features of the environment: firstly, the presence of an object in a particular radial direction, secondly, an estimate of the distance to that object, and thirdly, some information about the object’s 3D form. This was done by employing the stereo capability of the headphones (i.e. a pop sonified on the left or right corresponded to an obstructing object on the left or right of the participant), the time delay between the initiating click and the corresponding pop as with traditional echolocation (a long delay indicated that the object was further away from the participant than a shorter delay), and the volume of the pop sound itself. Importantly, participants were required to attend to not just a single particle’s pop, but the pops of all particles (of the 900) that have landed on virtual objects in front of the participant. The speed of the click and particle projection was chosen such that there was no temporal overlap between the pops corresponding to different clicks. In this way, a participant had to use the click and the collection of popping sounds to construct and continually update a distance-based map of his/her immediate (< 3m) surroundings in the VR environment. A video example of echolocation is provided here: https://www.youtube.com/watch?v=WFHEJ8pOego

#### 2.3.2 Distance-dependent hum volume modulation

Differing from the “snapshot” nature of the point-and-project simulated echolocation, the second sonification method we explored aimed to encode the spatial layout of objects using continuous audio beacons. Here, hums of different pitches were attached to different objects, and the volume of these hums was modulated based on a participant’s distance to the objects: shorter distances to objects correlated to their hums being louder. Furthermore, an object’s hum was only triggered when the participant entered a defined “humming zone” around the object, and as he/she moved closer to the object, the volume of the hum was linearly increased. A linear volume roll-off curve was selected over a log-based roll-off since it allowed changes in distance to an obstacle (given the bounds of its humming zone) to be more easily discerned. In the maze, the humming zone extended 1.2m from the wall (Fig 1A). In the obstacle corridor, the humming zone extended 3m radially from each obstacle, and 1.5m from the walls (Fig 1B). Using the stereo headphones, the panoramic position of the hum enabled participants to determine its direction and hence the spatial position of the obstacle.

We varied the types of humming sounds based on the objects emitting them. The walls in both the maze and the corridor were assigned a deep, resonant hum, while the obstacles in the obstacle corridor were each assigned a hum of unique pitch (to all the other obstacles and the walls). We opted for this approach because it was crucial for participants to be able to differentiate obstacles, especially when the humming zones of obstacles overlapped.

**Fig 3:**
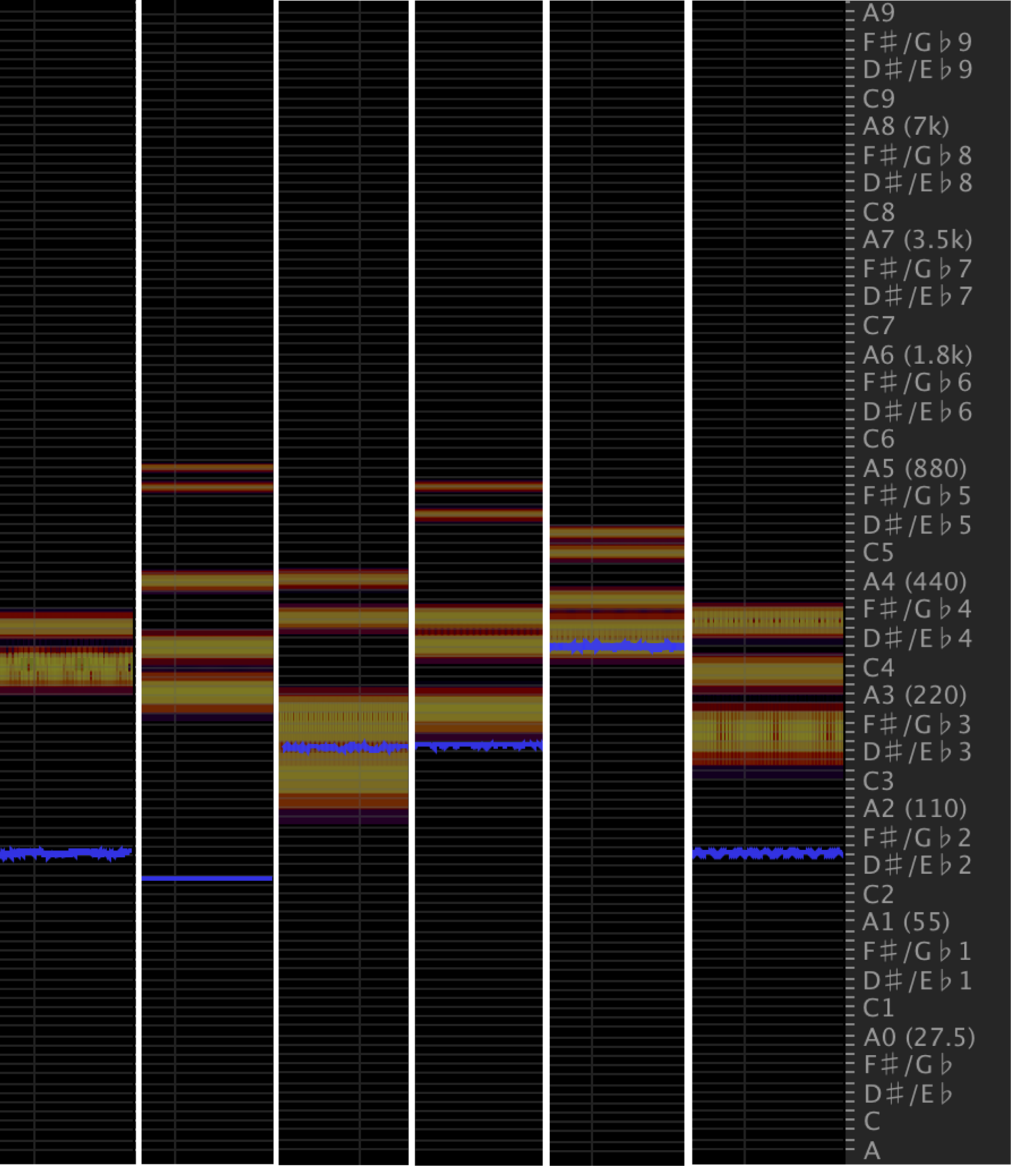
Spectral frequency displays of the additive sinusoidal hums used in the distance-dependent hum volume modulation sonification method. Each column (horizontal axis) corresponds to a hum, with its frequency components/notes shown on the vertical axis. The blue band indicates the hum’s core frequency, while the red and orange bands indicate other prominent frequencies.

Our rationale for choosing humming sounds over other types of sounds was two-fold: firstly, we wanted to create a *continuous* or smooth soundscape capable of capturing the presence and spatial location of multiple objects in an environment. The humming sounds were ideal for this since they could be looped seamlessly (compared to single beeps or pulses). Their continuous nature also allowed us to fuse audio representations across multiple objects in the environments. Secondly, we wanted to create an acoustically pleasant or harmonising soundscape with the motivation that our sonification mapping should be ambient and non-intrusive to users—essential in a real-world implementation of a SSD. For this reason, we selected hums since they have little temporal structure and are almost absorbed into the audio background unless specifically attended to. The hums themselves were additive blends of sinusoids, each of characteristic frequency, rather than pure tones which we viewed as harsher on the ear. The blends of frequencies were chosen based on what the researchers deemed to be pleasant sounding. Fig 3 summarises the core frequency components, or notes, of the hums used.

For brevity, we refer to this method of distance-dependent hum volume modulation as simply the humming sonification. A video example of humming is provided here: https://www.youtube.com/watch?v=aR5r10daK7Y

#### 2.3.3 Miscellaneous environmental sounds

In addition to the audio cues described above, the target/final location (represented by a golden star) emitted a continuous pinging sound in order to help participants localise the goal. The pinging had its volume modulated by a participant’s distance to it and the stereo headphones again enabled left/right differentiation of the star’s location. Additionally, at the end of each trial, to indicate that the goal had been reached, a completion sound (a jingle of chimes) was played.

### 2.4 Hardware & software

*Unity* (Unity Technologies, San Francisco, CA, USA), a 3D game engine, was used to create the virtual environments, and its built-in sound engine provided spatial audio features which were used to encode the environments using the two sonification methods. The spatial audio features allowed for the localisation of sound sources on the horizontal plane by taking the source and regulating the gains of the left and right ear contributions (relayed via the headphones) based on the distance and angle between it and the participant in the VR environment. The sonified environments were then deployed on a *Google Tango* tablet (Google, Mountain View, CA, USA). The tablet employs visual-inertial odometry using a 180° FOV fish-eye camera and its Inertial Measurement Unit (Fig 2B) to map its 3D position and rotation in real-time based on an initialisation point.

The software packages used for development included the *Tango Tablet Development Kit* (kernel version: 3.10.24-g36a1dd3; April 14, 2015), *Unity 5.0.1 Personal Edition* with C#, *Android SDK* tools (release 23.0.2 for Mac), and the *Tango Unity SDK* (release Ramanujan, version 1.17, July 2015). The *Tango* itself was running *Android KitKat* (version 4.4.2, API level 19).

Rather than using a keyboard or joystick-based spatial navigation paradigm, we aimed to create a fully immersive VR experience in which participants’ movements in the real world corresponded to their movements in the virtual world. To do this, the *Tango* was mounted in a *Durovis Dive 7* VR headset (Fig 2A). The headset allowed the device to be fixed to participants’ heads such that their 3D positional (translational) movement and 3D head rotation were tracked. The headset included a pair of lenses which projected the image on the *Tango* screen to a comfortable viewing distance for participants. The headphones (*Sennheiser HD 206 Stereo Headphones*) were wire-connected to the *Tango*. Importantly, the headphones were over-ear with a noise attenuation feature, thus ensuring that any external sounds from the environment were largely suppressed.

### 2.5 Experimental protocol

Testing was carried out in a large indoor hall or a large outdoor space, thus comfortably fitting the virtual environments and reducing the risk of participants colliding with physical objects. The physical size of the hall was 20 × 25 metres. The outdoor space was a flat manicured lawn, the weather was mild, and no other people, besides the experimenter and the participant, were present. The testing location was selected based on the availability of the hall, but also allowed the robustness or generalisation of our methods to be tested in two different environments. Of the 18 participants, 5 were tested outdoors, and no difference in navigational performance was noted.

Six experimental conditions were undertaken by each participant. Each condition included a minimum of six repeated trials. Participants were instructed to reach the star goal as quickly as possible while avoiding walls and/or obstacles. Collisions with either were conveyed to the participant by the *Tango* vibrating gently. This vibration was intended to notify participants of a collision and, without being too noxious, to motivate them to avoid collisions as much as possible. Trials were discarded from analysis if 1) the *Tango* tracking was lost due to a device or initialisation failure, or 2) the participant failed to reach the goal within 150 seconds. Out of the total 641 valid trials across the 18 participants and 6 conditions (visual, humming and echolocation in both environments), 39 of these trials were discarded. Of these 39 trials, 28 were discarded due to device/initialisation failure, while the remaining 11 were discarded due to a participant taking over 150 seconds to reach the goal. The distribution of the 11 no-goal-reached trials was as follows: [5, 1, 1, 2, 2, 0] for the first to sixth trial, respectively.

**Stage 1: Visual-only condition** A purely visual condition was conducted to familiarise participants with both of the virtual environments and to determine a visual baseline for task performance. The virtual environments were visible on the *Tango* screen mounted in the *Durovis* headset and participants were instructed to walk toward the star at a natural walking pace while avoiding walls and/or obstacles. The star emitted a ping that served as an audio beacon, and played the completion chime when reached. No other sounds were present. Six trials of the visual-only condition in each environment (maze and obstacle corridor) were conducted with the order of environments randomised across participants.

**Stage 2: Spatial audio training** Following the visual-only condition, an opaque piece of cardboard was taped over the screen of the *Tango* for the remainder of the experimental conditions. Henceforth, only audio information was available to participants via the headphones. In order to acquaint participants with the concept of spatial sound, a training stage was conducted: participants were instructed to walk toward a pinging goal positioned 13 metres directly ahead of them in an obstacle-free environment. Training was continued until participants were able to comfortably localise the goal using the stereo and distance-based volume changes of the pinging sound. This did not prove to be too difficult, with all 18 participants doing one training run, 10 doing a second, and 3 doing a third.

**Stage 3: Sonification conditions** Six trials of each of the two sonification conditions were conducted, with a familiarisation period for each done prior to the test trials. The testing protocol was laid out as follows:

- *Echolocation*: a familiarisation period followed by 6 trials done in the maze and another 6 trials done in the obstacle corridor.
- *Humming*: a familiarisation period followed by 6 trials done in the maze and another 6 trials done in the obstacle corridor.

The order of the echolocation and humming sets was randomised across participants, as was the order of the environments tested within each set.

The familiarisation period for each sonification condition was conducted in a simplified version of the obstacle corridor set-up: a single obstacle was located centrally at 4m away from the starting point. In the echolocation training period, this obstacle and the walls received and projected the echolocation click and popping sounds as described in Section 2.3.1. In the humming training period, objects hummed depending on the participants’ distance to them as described in Section 2.3.2. Familiarisation was considered complete when both the participant and the experimenter agreed that the participant understood the task aims and sonification rules. This proved to be a relatively short period: for echolocation training, all 18 participants did one training run, 13 did a second and 3 did a third, while for humming training, all 18 participants did one training run, 13 did a second, 2 did a third and 1 did a fourth. A two-sample *Kolmogorov-Smirnov* test does *not* reject the null hypothesis (*p* = 1) that the number of training runs for each sonification condition comes from the same distribution. This suggests no significant difference in training duration between the two sonification conditions.

### 2.6 Experimental metrics

The 3D location (x, y, z) and 3D head rotation (yaw, pitch, roll) of the participant was recorded from the *Tango* at a rate of 2Hz. From these data, participants’ 6D path trajectories through each of the environments in each of the conditions were reconstructed. Data were written to a text file on the *Tango* at the completion of each trial and later processed with *MATLAB* (MathWorks, Natick, MA, USA) using custom scripts. Analysis included looking at the “bird’s eye view” path of participants, the length of this path, and participants’ instantaneous, mean and peak velocities. Measures designed to probe visual awareness and navigational strategies were additionally investigated through the derivation of 1) instantaneous and mean head rotation angle, and 2) deviation distances from obstacles and walls, both of which are expanded on below.

**Head rotation angle.** Since sound in the virtual environments was fully stereo, an indicator of exploration was taken to be the amount of lateral head rotation that participants made whilst navigating the virtual environments. Given that the *Tango* was head-mounted, the *Tango*’s rotation corresponded to participants’ head rotation. The Euler angle for rotation about the vertical axis (yaw) was extracted at each time point and compared to the instantaneous path angle (that is, the direction of walking calculated using the positional data). The difference in angle was then transformed such that a left head rotation fell in the range from 0° to −90°, and a right head rotation fell in the range from 0° to +90°. The mean left and right head rotation was calculated per trial, and their absolutes summed to give a total head rotation value between 0° and +180°.

**Deviation distances.** Detection distance may be defined as the distance at which a participant explicitly identifies an obstacle ahead of them [80]. One proxy for detection distance is a subject’s “deviation distance”, the distance of a participant to an obstacle when he/she begins to adjust his/her trajectory to avoid it. This value was calculated by extrapolating participants’ path trajectories, determining if their trajectories intersected with the area occupied by obstacles at each time point, and if so calculating the distance at which participants deviated from this “collision course” [78]. In the corridor environment, this calculation was applied to the five columnar obstacles. In the maze, however, it was necessary to reformulate the calculation of deviation, and a midline deviation was used instead. The midline path, considered the optimal path, is the path equidistant to all walls at any given time point. At each time step, the perpendicular distance of a participant’s path trajectory to this midline was calculated, and the means of the deviations left and right of the midline found. In the visual maze condition, participants often “clipped” the corners, and for this reason the corners of the maze were not included in the midline deviation calculation.

### 2.7 Statistical analysis

ANOVA analyses were performed using *SPSS* (IBM Corp., Armonk, NY, USA) to investigate the relationship between experimental condition and the above-mentioned metrics across participants. To account for the across-trial variability of each participant within a given condition, the mean over their six trials for each measure was calculated, and these values averaged across participants. Additionally, because the maze and the obstacle corridor are fundamentally different, with performance in each not necessarily comparable, the analysis was split accordingly (see Section 3.1 and Section 3.2). Results are reported as mean ± standard error, with asterisks used to indicate statistical significance as follows: *p* < .05(*), *p* < .01(**) and *p* < .001(***). The *p*-values in pairwise comparisons for multiple comparisons were corrected using the Sidak correction. Greenhouse-Geisser estimates were used to correct for violations of the assumption of sphericity in the results.

## 3 Results

### 3.1 Maze

**Fig 4:**
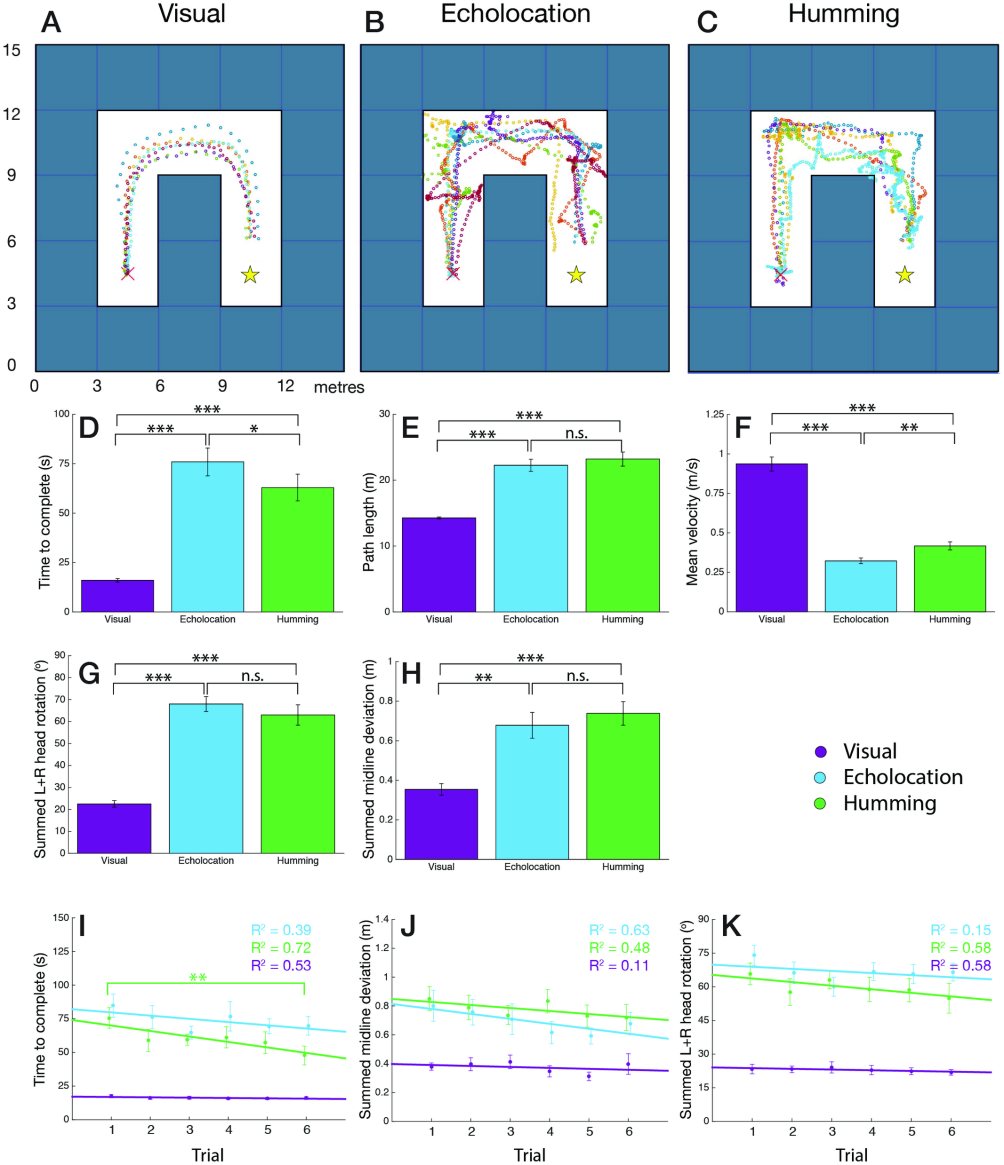
Maze navigational behaviours and results. (A, B, & C) Examples of participant trajectories through a maze, using visual-only cues (A), echolocation audio-only cues (B), and humming audio-only cues (C). (D, E, F, & H) Basic efficiency and strategy of navigation through the maze in the visual (purple), echolocation (blue) and humming (green) conditions: time to reach goal in seconds (D), path length in metres (E), mean velocity in metres per second (F), and summed left and right midline deviation in metres (H). Error bars indicate standard error. Statistical significance indicated as follows: not significant (n.s.), *p* < .05(*), *p* < .01(**) and *p* < .001(***). (G) Summed left and right head rotation in degrees. Head rotation from head straight (0°) direction, comparing visual (purple), echolocation (blue) and humming (green) conditions in the maze. Error bars indicate standard error. Statistical significance indicated as follows: not significant (n.s.), *p* < .05(*), *p* < .01(**) and *p* < .001(***). (I, J, & K) Learning curves over 6 trials comparing visual (purple), echolocation (blue) and humming (green) conditions in the maze: time to complete in seconds (I), summed left and right midline deviation in metres (J) and summed left and right head rotation in degrees (K). Linear regression line with *R*^2^-statistic shown for each condition in corresponding colours. Error bars indicate standard error. Significance symbols mark significance of effect between first and sixth trial. Statistical significance indicated as follows: not significant (not shown), *p* < .05(*), *p* < .01(**) and *p* < .001(***).

Participants were able to navigate the maze environment in both visual and sonification conditions. At the start of each trial, participants were placed equidistant from the walls and forward-facing at the starting point. By nature of the maze and sonification set-up, this meant that participants received no audio information if they did not move from this point and gained no extra benefit/advantage by remaining there. We therefore formally began each trial when the participant began walking (we took this to be when their walking speed increased above 20% of their peak-to-peak velocity for that trial). Across participants, this start-delay was 2.42s with echolocation and 2.28s with humming, a statistically insignificant difference between the sonifications. Fig 4A–4C show the path trajectories of participants for the visual, echolocation and humming conditions, respectively.

#### 3.1.1 Efficiency of maze navigation

We first investigated participants’ ability to navigate without making mobility errors (i.e. collisions). Total collisions were tallied over the 6 trials per participant. A significant effect is seen between conditions (*F*(2, 34) = 8.906, *p* = .001): as expected, the visual condition (0.06 ± 0.06 collisions) outperforms the sonification conditions (echolocation: 4.44 ± 1.12 collisions; humming: 4.78 ± 1.22 collisions).

Beyond number of collisions, the time to goal, path length and mean velocity reveal more about the ease with which participants navigated through the maze (Fig 4D–4F). In the visual condition, time to complete (16.24 ± 0.84s), path length (14.91 ± 0.46 m) and mean velocity (0.96 ± 0.05 m/s) were all significantly different from the same metrics in each sonification method: echolocation with 73.81 ± 6.39s (*p* < .001), 22.78 ± 0.90m (*p* < .001) and 0.34 ± 0.02 m/s (*p* < .001), and humming with 60.77 ± 6.15s (*p* < .001), 23.47 ± 0.97m (*p* < .001) and 0.44 ± 0.03 m/s (*p* < .001), respectively. Pairwise comparisons show no significant difference between sonification methods for path length (*p* = .833) however, a significant difference is noted in the time to complete (*p* = .049) and mean velocity (*p* = .001) achieved with echolocation versus humming. The higher humming mean velocities suggest that participants moved faster through the humming maze. Despite this, the paths taken in each sonification condition were still approximately 60% longer than in the visual condition, indicating that participants were not taking the most efficient path and were more exploratory/cautious in their maze navigation. This is supported by the longer times to reach the goal and the lower mean speeds compared to the visual condition (Fig 4D–4F).

A further point to draw from Fig 4D–4F is the robustness of the visual control: the *Tango* was able to accurately register real-world distances travelled. Given that the blocks constituting the virtual maze were 3 × 3 × 3m in real-world size and that every path to goal was standardised to 7 block steps in length (with the end of the trial triggered when the participant came within 1.75m of the goal), the midline path length is 16.25m. The mean path length of participants was just under 16.25m, explained by the fact that often participants clipped the corners in the visual condition. Also bolstering the visual control is participants’ mean velocity of ∼1m/s which is close to the average human walking speed of 1.4m/s [81]. This point suggests that participant performance in the visual control approaches normal (non-virtual) visual performance.

Beyond the above-mentioned basic metrics, we also explored the more nuanced navigational behaviours of participants. Specifically, we hypothesised that participants may be adopting a “follow the wall” strategy in the maze, whereby by maintaining a constant distance to either the left or right wall (detected by a constancy in their audio cues, whether echolocation or humming) participants would be able to eventually reach the goal. To investigate this, we computed the mean left and right deviations from the central maze midline per trial. Fig 4H shows the sum of the absolute left and right midline deviations for the visual (0.37 ± 0.03m), echolocation (0.70 ± 0.06m) and humming (0.78 ± 0.06m) conditions. A significant main effect of condition on the midline deviation is noted (*F*(2, 34) = 20.519, *p* < .001) with pairwise comparisons between the visual and echolocation condition (*p* = .001) and the visual and humming condition (*p* < .001) suggesting that without sight, participants deviate further from the midline than with sight.

#### 3.1.2 Head rotation

The usefulness of a head rotation was hypothesised to differ for the two sonification conditions. With echolocation, a turn of the head changed the direction of the particle projection. This offered participants new information about the environment within their new FOV. Humming, on the other hand, benefited differently in that a head rotation offered a change in stereo of the hum, but only when a participant was within the humming zone of an object. A head rotation toward an object when outside its humming zone, for example, did not trigger its humming.

The mean left and right head rotation was calculated per trial (0° straight ahead, −90° maximum left rotation, +90° maximum right rotation), and their absolutes summed to find a total head rotation per trial between 0° and 180° (Fig 4G). We expected that in the visual condition simple shifts in gaze rather than full head turns would be sufficient for navigating the maze. In line with this hypothesis, the visual condition saw only 22.93 ± 1.39° of total head rotation and this was significantly different (*p* < .001) from that in the echolocation (66.64 ± 3.14°) and humming (60.12 ± 4.54°) conditions. For the reasons described above, we also hypothesised that in the humming condition, a head rotation would be less useful than in the echolocation condition, however, pairwise comparisons showed no significant difference in total head rotation between the two sonification methods (*p* = .303).

#### 3.1.3 Maze learning rates

The efficiency of participants’ maze navigation improved over trials and since with each trial the maze path was randomised, the improved proficiency suggests that participants were increasing their understanding of each sonification method. To show this, a one-way repeated-measures ANOVA was performed on trial within each condition.

Fig 4I–4K show the learning curves over 6 trials for time to complete, summed midline deviation, and summed head rotation. Improvements were not expected in the visual condition and this was indeed the case, with no significant effect of trial observed for any of the three metrics in the visual condition. In the echolocation condition, no significant (*p* = .145) improvement was seen in time to complete (trial 1: 84.69 ± 8.65s to trial 6: 69.69 ± 6.93s) nor for summed midline deviation (*p* = .386) or summed head rotation (*p* = .051). However, in the humming condition there was a significant (*p* = .008) improvement over the six trials in time to complete (trial 1: 75.56 ± 7.61s to trial 6: 47.7 ± 7.00s). Summed midline deviation and summed head rotation did not significantly change in the humming condition over trials. Overall, even if statistical significance was not always achieved, all metrics showed improving trends for the sonification conditions.

### 3.2 Obstacle corridor

**Fig 5:**
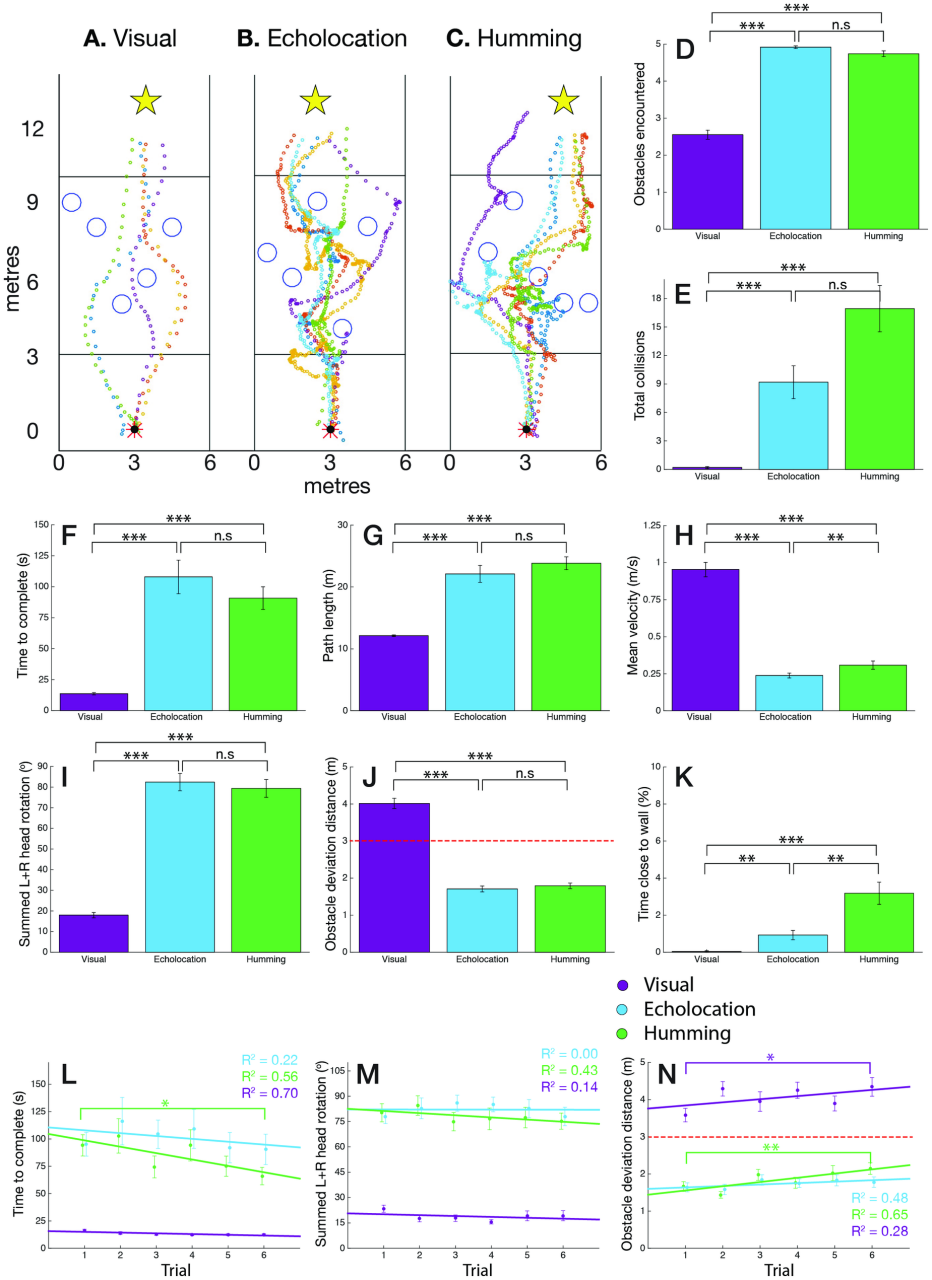
Obstacle corridor navigational behaviours and results. (A, B, & C) Examples of participant trajectories through particular obstacle corridor arrangements using visual-only cues (A), echolocation audio-only cues (B), and humming audio-only cues (C). (D, J, & K) Active interaction with obstacles in the corridor in the visual (purple), echolocation (blue) and humming (green) conditions: number of obstacles encountered at least once per trial (D), obstacle deviation distance (J), and percentage of obstacle patch time per trial spent <0.4m from left or right wall (K). Red dashed line shows 3m echolocation and humming threshold. Error bars indicate standard error. Statistical significance indicated as follows: not significant (n.s.), *p* < .05(*), *p* < .01(**) and *p* < .001(***). (E, F, G, & H) Basic efficiency of navigation through the obstacle corridor in the visual (purple), echolocation (blue) and humming (green) conditions: total number of collisions over 6 trials per participant (E), time to reach goal in seconds (F), path length in metres (G), mean velocity in metres per second (H). Error bars indicate standard error. Statistical significance indicated as follows: not significant (n.s.), *p* < .05(*), *p* < .01(**) and *p* < .001(***). (I) Summed left and right head rotation from head straight (0°) direction in the corridor in the visual (purple), echolocation (blue) and humming (green) conditions. Error bars indicate standard error. Statistical significance indicated as follows: not significant (n.s.), *p* < .05(*), *p* < .01(**) and *p* < .001(***). (L, M, & N) Learning curves over 6 trials comparing visual (purple), echolocation (blue) and humming (green) conditions in the obstacle corridor: time to complete in seconds (L), summed left and right head rotation in degrees (M), obstacle deviation distance in metres (N). Red dashed line shows 3m echolocation and humming threshold. Linear regression line with *R*^2^-statistic shown for each condition in corresponding colours. Error bars indicate standard error. Significance symbols mark significance of effect between first and sixth trial. Statistical significance indicated as follows: not significant (not shown), *p* < .05(*), *p* < .01(**) and *p* < .001(***).

Compared to the maze, the obstacle corridor offered an environment much closer to a possible real-world scenario. The obstacles, by way of the randomness of their positioning, made for a far less predictable path to the goal compared to the mazes, with multiple possible paths available. As with the maze, each trial was formally started when participants’ walking speed increased above 20% of their peak-to-peak velocity for that trial. Across participants, this start-delay was 2.07s with echolocation and 2.18s with humming, again a statistically insignificant difference. For illustration purposes, Fig 5A–5C show the path trajectories of participants in selected corridor arrangements.

#### 3.2.1 Efficiency of obstacle corridor navigation

As with the maze, the number of collisions with both walls and obstacles in the corridor was used as an indicator of navigational efficiency (Fig 5E). Like the maze, condition had a significant main effect on total collisions (*F*(1.622, 27.566) = 21.36, *p* < .001; sphericity *χ*^2^(2) = 6.342, *p* = .042 corrected with Huynh-Feldt estimates) with a significant difference observed between the visual condition (0.17 ± 0.09 collisions) and each sonification method (echolocation: 8.33 ± 1.64, *p* < .001; humming: 15.50 ± 2.37, *p* < .001). No statistically significant difference was noted between sonification methods (*p* = .067) however, by inspection it can be seen that a mean total of 7 more total collisions over the six trials occurred in the humming obstacle corridor compared with the echolocating obstacle corridor (Fig 5E). This suggests that humming was not as effective at signalling an imminent collision.

As before, time to complete, path length and mean velocity revealed further dynamics of the obstacle corridor navigation (Fig 5F–5H). Condition had a significant effect on all three metrics due to the strong performance of participants in the visual condition. For time to complete (Fig 5F), the visual condition, taking 13.31 ± 0.82s, was significantly faster than the echolocation (101.89 ± 12.68s, *p* < .001) and humming (84.74 ± 9.09s, *p* < .001) conditions. A pairwise comparison between echolocation and humming showed no significant difference (*p* = .537). Path length yielded no interesting differences other than a shorter path length in the visual condition (12.12 ± 0.12m). Mean velocity was significantly affected by condition (*F*(1.308, 22.239) = 207.73, *p* < .001): significant pairwise differences were seen between the visual condition (0.98 ± 0.05m/s) and each sonification condition (echolocation: 0.25 ± 0.02m/s, *p* < .001; humming: 0.32 ± 0.03m/s, *p* < .001), respectively. Further, a significant difference was noted between the mean velocities of the two sonification conditions (*p* = .008), indicating that, like in the maze, participants moved at higher speeds when using the humming sonification.

The deviation distance was computed for each columnar obstacle encountered in the obstacle corridor (see Section 2.6). Condition had a significant main effect on the obstacle deviation distance (*F*(2, 34) = 172.712, *p* < .001) with participants deviating from obstacles at 4.06 ± 0.13m in the visual condition, but only at 1.73 ± 0.08m and 1.82 ± 0.07m in the echolocation and humming conditions, respectively (Fig 5J). Pairwise comparisons show the difference between the visual and each sonification condition is significant (*p* < .001) but not between echolocation and humming directly (*p* = .753). As expected, with sight participants gave obstacles a wide berth, whereas with only audio, participants moved closer to obstacles, which also resulted in a higher number of collisions. It is worth mentioning that obstacles only began to echolocate/hum at 3m which placed an upper limit on the achievable deviation distances in the sonification conditions (shown by the dotted red line in Fig 5J).

The obstacle deviation metric does not account for the portion of time per trial spent actively encountering the five obstacles. Given their random positioning, it was possible that a participant spent more time encountering obstacles in one trial than in another. In addition to this, the identifiable deep hum of the walls could lead participants to avoid the majority of the obstacle segment by simply walking against either one of the walls. To quantify this Fig 5D shows the number of obstacles (of the total 5) actively encountered per trial. An obstacle was considered to be actively encountered if a participant walked within the audio zone/threshold of that object. Repeat encounters were not factored in, although were likely common. In the visual condition, only 2.54 ± 0.13 obstacles were encountered, whereas 4.88 ± 0.42 and 4.72 ± 0.07 obstacles were encountered in the echolocation and humming conditions, respectively. Additionally, since, in the corridor, all obstacles were positioned within a 6 × 7m obstacle patch (see Fig 1B and 1E), we could compute the percentage of the time per trial spent in the obstacle patch in which the participant was less than 0.4m from the left or right wall (Fig 5K). All conditions show values of less than 3% (visual: 0.03 ± 0.03%; echolocation: 0.92 ± 0.24%; humming: 2.95 ± 0.56%). These results together suggest that in each sonification condition participants spent a large portion of the time per trial in the obstacle segment actively encountering and avoiding obstacles.

#### 3.2.2 Head rotation

As with the maze, it was also relevant to look at the use of head rotation in the obstacle corridor. Condition had an effect on summed left and right head rotation (*F*(2, 34) = 128.32, *p* < .001) with the visual condition using a significantly smaller amount of head rotation (18.82 ± 1.63°) compared to the echolocation (82.19 ± 3.97°) and humming (77.70 ± 4.33°) conditions. The difference between the visual and each sonification condition was significant (*p* < .001) but was not significant between sonification conditions (*p* = .754) (Fig 5I).

#### 3.2.3 Obstacle corridor learning rates

Given the corridor’s similarity to navigating a cluttered indoor environment, it was particularly interesting to analyse learning rates in this scenario. Just as with the maze, a one-way repeated-measures ANOVA was performed on trial within each condition.

Fig 5L shows that while the time to complete remained largely consistent from trial 1 to 6 in the visual (16.11 ± 1.24s to 12.44 ± 0.75s) and echolocation (95.25 ± 10.91s to 90.53 ± 13.91s) condition, it dropped some 30 seconds for the humming condition (94.14 ± 9.76 to 66.00 ± 7.95s; *p* = .029). The faster times after 6 test trials of humming imply a degree of learning and improved efficiency. Trial had no significant main effect on summed left and right head rotation within each condition, with only a slightly reduced use of humming head rotation observed over the six trials. This suggests that within 6 trials, participants were not varying their use of head rotation, for example as a strategy for navigation (Fig 5M). Obstacle deviation distance (Fig 5N) also showed significant improvement with trial in the humming condition (*F*(5, 75) = 4.021, *p* = .003), but was not significant in the echolocation condition (*F*(5, 80) = 0.731, *p* = .602). Both, however, showed overall increasing trends in deviation distance from trial 1 to 6 (*R*^2^ = 0.652 and *R*^2^ = 0.482 for humming and echolocation, respectively) indicating that with more practice, participants were able to more efficiently “weave” within safer distances between the obstacles in the corridor.

### 3.3 Qualitative experience

**Fig 6:**
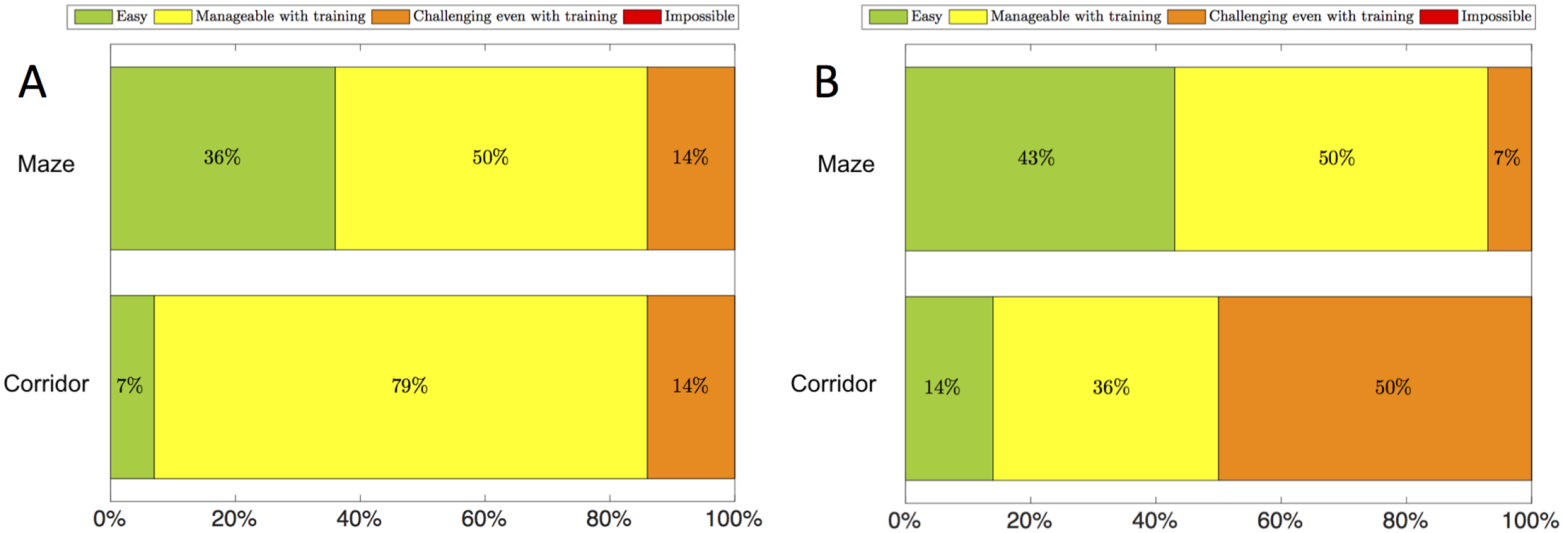
Qualitative participant feedback. (A)“How easy/intuitive did you find the echolocation sonification in each environment?” (B)“How easy/intuitive did you find the humming sonification in each environment?” Green: easy. Yellow: manageable with training. Orange: challenging even with training. Red: impossible.

A final component enhancing the quantitative results presented above is the qualitative feedback received from participants following their two testing sessions. Since the spread of SSD naivety and gaming experience across participants was small, it was not expected to be correlated with participant performance. Looking beyond this, it was informative to look at participants’ overall experience with each sonification method, and its ease or intuitiveness in each environment. Fig 6A and 6B show the ratings received from 14 out of 18 participants when asked about the intuitiveness of each method. The maze had a largely even spread across ratings from “easy” to “challenging even with training”, with 36% and 43% of participants finding it easy for echolocation and humming, respectively. A common comment was that the mazes were more basic since one simply needed to walk in straight lines. One particular participant whose performance was notably poor in the mazes across both sonification conditions commented that her spatial memory and internal orientation were poor, and this made it difficult for her to spatially connect the corridors she was exploring. As a result, after a period of hesitation/turning/head rotation, she often began to navigate down a corridor from which she had just come. The obstacle corridor, on the other hand, had a more distinct qualitative split, with echolocation perceived as manageable with training by 79% of participants, while humming was perceived as such by only 36% of participants, with 50% rating it as challenging even with training. The *perceived* ease/intuitiveness of the sonification methods, therefore, contradicts participants’ true navigational performance using them in the corridor environment. These results also suggest that of the two environments, the maze was easier to navigate, likely because of its limited layouts, while the obstacle corridor posed a bigger challenge. It should be noted that no participant rated either sonification method as “impossible”.

## 4 Discussion

The principal outcome of this work is that participants without access to sight were able to navigate a virtual space and detect and avoid obstacles using our two novel sensory substitution sonification approaches. Furthermore, they learned to do so with a small amount of training (less than 3 hours) and minimal instruction. Our results are promising in the view of using these sonification strategies to help people with visual impairments to navigate real world environments. Moreover, the self-locomotion-guided virtual reality environment we developed to test the utility of our stereosonic vision mappings provides a realistic yet safe, controlled, and flexible paradigm for testing navigation and mobility skills, and allows for the automatic extraction of many useful performance metrics.

### 4.1 Development of an experimental paradigm to test sonification mappings

In this work, we successfully built a visual-to-audio SSD using a head-mounted *Tango* tablet and a pair of stereo headphones. We established an experimental testbed for the device by using standardised metrics to analyse participants’ detailed navigational behaviour through randomly generated VR environments. Participants’ navigational data was obtained by extracting the 3D positional and 3D rotational tracks of a head-mounted *Tango* tablet. These tracks corresponded to participants’ body position and head rotation at fine-grained time steps over the length of each trial. This testbed allowed us to successfully measure the utility of two sonification methods for the task of spatial navigation.

Our VR experimental paradigm provides important improvements over keyboard- and joystick-based paradigms which to date have been ubiquitous in studies on spatial navigation [82, 83], capturing the important contribution of proprioceptive feedback to VR navigation and mobility [72, 73]. Importantly, this was achieved using a portable, inexpensive tablet that can be used in stand-alone fashion in any sufficiently large open space, and did not require the large amount of dedicated infrastructure of previously published locomotion-controlled auditory VR [68, 74–76]. This makes locomotion-controlled, multi-sensory immersive VR accessible to smaller, independent research groups, or those who do not use immersive VR as their principal methodology.

Like other VR paradigms, our VR paradigm lends itself well to the automatic and randomised generation of simulated environments, and avoids real-world mobility hazards to participants. Moreover, the continuous tracking of real walking behaviour in the virtual world allows for the automated extraction of measures that capture the dynamics of walking and navigating, such as path length, speed, head rotation, and deviation distances from objects. In the current study, we generated simplified models of real-world scenarios, a necessary first step, however the flexibility of the experimental paradigm will enable the testing of audio-only navigation in more complex virtual environments in the future, for example multi-room scenarios with objects of varying shapes and sizes.

While we have used this navigation testing paradigm to investigate the utility of our sonification mappings, the paradigm would be equally suited for testing navigation using existing SSDs. Directional spatial-to-audio SSDs like the EyeCane have already been successfully tested in VR environments [51, 84, 85], so it would only require the use of a tracking device such as the *Tango* used here to convert this into a locomotion-based VR paradigm. To test realistic echolocation-type SSDs [59, 61–63] in VR, the most important additional requirement would be the accurate modelling of acoustics in the virtual environment to accurately render the temporal and spectral information present in real-world echoes [86], something that has already been achieved in this specific context [60, 87]. Finally, to test navigation in SSDs that take 2D images or videos as input, whether auditory such as the *vOICe* or EyeMusic [42, 43], or tactile such as [37–41], the image of the VR environment as rendered on screen can simply be used as a substitute to the regular video input of such SSDs.

Additionally, the wealth of data on the dynamics of navigation automatically extracted from the tracking device and its lightweight, low-cost control via real locomotion opens up other exciting use cases. We believe this approach will not only prove useful for studies of mobility and sensory substitution, but also for studies of navigation and spatial cognition where a contribution of proprioceptive information is relevant; for instance, in immersive studies of spatial learning and memory [88, 89] and how they may be affected by clinical conditions such as Alzheimer’s Disease [90, 91] or depression [92].

### 4.2 Comparison of stereosonic navigational behaviour between sonification mappings

**Visual/sighted control.** Participant walking velocity in the visual control condition approached 1m/s, close to the average human walking speed of 1.4m/s [81]. These measurements verify the robustness of the visual control against which performance in each of the sonification conditions is compared.

**Simulated echolocation.** The emitted click and distance-dependent “pops” of simulated echolocation necessitated the following: firstly, participants needed to sample their surroundings by orienting their head and secondly, using the returning waves of pops, participants needed to stitch their audio-based representations together in order to construct a representation of the full scene. For these reasons, echolocation is unlike the more passive humming sonification method, and more like the task of looking and “seeing”. By comparing participant performance, however, the simulated echolocation sonification appeared to be less intuitive than the humming sonification. This was supported by overall slower echolocation navigation speeds in both the maze and the obstacle corridor, indicative of participants being more hesitant. This hesitancy could have been the result of a number of factors. Firstly, compared to the humming sonification, there is a higher cognitive load in processing the click-pops of echolocation. Participants reported that the click-pop delays were often difficult to interpret, however, with more training, they said they may have been able to draw more information from them. A second reason for higher hesitancy with simulated echolocation is the potential limitation imposed by its update speed. The click rate (every 1.75s) placed an upper bound on the speed at which participants received an up-to-date acoustic snapshot of the environment. It is possible that with a higher click frequency, participants would have been able to move more quickly, however, this could come with the risk of them losing the ability to temporally discriminate pops if they scattered too quickly.

Across both environments a unifying strategy for simulated echolocation was hypothesised to be one in which the “quietest” path or the path of least resistance was followed. In other words, if no obstacles were directly in front of a participant, then the returning pop sounds were few and so the soundscape relatively quiet. In this way, a path could be weaved between obstacles. Consistent with this, there was a trend towards fewer collisions in the echolocation condition compared to humming (by 46%, p = 0.067).

**Distance-dependent hum volume modulation.** Participant task performance indicated that encoding objects’ spatial distance using volume-modulated humming was an intuitive sonification method, more so than the simulated echolocation. In both the maze and obstacle corridor, navigation using the humming sonification was faster (higher mean velocity and lower time to reach the goal) compared to the simulated echolocation along equivalent path lengths. This suggests higher confidence levels with the humming technique. Looking at the more challenging task of obstacle avoidance in the corridor environment, with humming, the walking speeds were on average 27% slower than those in the maze. Although the humming obstacle corridor velocities were faster than those in the echolocating obstacle corridor by 0.7m/s, the drop in speed from humming-based navigation in the simple maze to the more complex obstacle corridor was larger than the corresponding performance drop using echolocation-based navigation.

These results together suggest that of the two sonification methods, humming was quicker and easier to learn than echolocation. Humming, however, is inherently limited by the amount of information it can represent. A hum, based on its volume level conveys distance to an obstacle, and based on its stereo components conveys information about its spatial position. On the other hand, a hum offers no information about object shape. Furthermore, absolute hum volume, necessary for participants to ‘calibrate’ their volume-distance correspondence, may be difficult to immediately discern. Critically too, the presence of multiple obstacles, resulting in the overlapping of humming zones, was reported to make it challenging to distinguish space between obstacles or clear paths ahead. We believe, therefore, that simulated echolocation has greater potential for representing more complex and detailed environments for the task of navigation. Pointing toward this, participants rated echolocation as easier than humming in the more complex corridor environment in their follow-up qualitative feedback, despite their poorer navigational performance here. One possible explanation for this difference could be that participants are positively projecting their capacity to learn a richer sonification for more complex environments in the future (i.e. perceiving the method as easier in the present because of their predicted ability to improve or master the method in the future).

To enable the estimation of distance with a humming approach, future work could include modulating the hums in ways that would make judging absolute distance easier for the human auditory system. Hums could be pulsed with a frequency proportional to obstacle distance, or modulated in pitch or with a filter envelope as a participant approaches an obstacle. The presence of multiple objects as overlapping sources of hums also poses a challenge, as it is easy to overwhelm a participant with a cacophony of sound. Here, an intelligent way of identifying the most mobility-relevant objects in the scene might be useful so that sounds from other less relevant objects or objects further away may be suppressed.

### 4.3 Observed learning effects in stereosonic navigation

For each sonification condition, participants received minimal instruction prior to the trials and no feedback during the trials. As part of the minimal instruction, participants underwent a training period with the device—this took place in a drastically simplified version of the obstacle corridor (with only one obstacle) and comprised only a small number of trials. The training period was primarily intended to familiarise participants with the headset and experimental procedure rather than the sonification methods themselves, and the number of training trials did not differ between conditions. The brevity of the instruction and training period was also intended to provide a fair baseline for comparison to other visual-to-audio SSDs in navigational proficiency.

With this in mind, it is therefore impressive that learning effects were observed for the humming sonification over only 6 trials. The clearest learning effect was seen in the decrease in time taken to reach the goal: improvements of 24.2s and 28.1s were achieved in the maze and the obstacle corridor, respectively. Furthermore, in the obstacle corridor environment, obstacle deviation distances improved by ∼0.5m using the humming sonification. No significant effect was seen for echolocation, though the direction of the effects was consistent with improvement.

While the sonification performance does not reach performance when sight is available, the results speak to the possibility that with further training this performance difference could be reduced. More experience with each sonification method as well as a more detailed understanding of the sonifications’ formulations (in particular simulated echolocation which shows promise in representing more complex environments) may allow participants to narrow this performance gap with practice.

### 4.4 Considerations for real-life application

From the current study it is too bold to claim that the two sonification methods explored here encode visual spaces in sufficient detail for seamless spatial navigation. Our results, however, do suggest that participants were able to obtain a 3D spatial awareness of their virtual surroundings. The fact that participants were able to spatially place themselves and manoeuvre through two distinct virtual environments, avoiding walls and simple obstacles, and also improve their navigational performance over trials is indicative of this. We believe that it is, therefore, interesting to consider the potential for these strategies as a real-life sensory substitution device for visually impaired people.

To this effect, it will be necessary to investigate how blind or visually impaired participants specifically adapt to these different sensory substitution approaches, and how the approaches complement or interact with their existing navigation strategies. On one hand, blind or visually impaired participants may in fact outperform our normally-sighted participants, as it has been shown that early-blind individuals in particular may display superior performance in auditory tasks like pitch discrimination [93] and spatial sound localisation [94–97]. In addition, they will be more experienced in using such spatial properties of audio signals to navigate and detect obstacles in their environment [32, 33]. With regard to our hypothesised audio-based navigational strategies, blind or visually impaired individuals might in fact be more inclined than our sighted participants to use head rotation for echolocation as they will know from experience that this helps to sample the auditory environment.

Conversely, in particular those visually impaired participants who have been blind from birth or a very young age may have altered representations of locomotor space that could interfere with performance [30, 98]. This may reflect a greater tendency to rely on an egocentric rather than allocentric spatial reference frames [99], which is thought to be particularly pronounced for larger-scale spatial representations such as those relevant to locomotor activity [100]. There is evidence that such altered spatial representations may negate the advantages of superior auditory discrimination in sound localisation in larger spaces [101]. How well the performance of our sighted participants correlates to the performance of blind and visually impaired individuals is therefore an important question to address in future work.

Moreover, in order to translate our sonification mappings to the real world, it will be necessary to implement the current virtual world sonification in real 3D space, and ultimately transfer the mappings onto a stand-alone wearable SSD. By pairing an RGB camera with a depth sensor and applying computer vision algorithms to the incoming video streams, it is possible to extract 3D spatial information of the real-world environment. On the incoming RGB images, object detection methods [102] will localise the presence/absence of objects, and object recognition methods [103, 104] will identify the *types* or classes of these objects (for example, desks, chairs, people, but also more abstract categories like walls, floors and ceilings). The incoming depth maps (from the depth sensor) will provide real-world distance estimates from the camera to densely scattered points in the environment. If the camera were mounted on the user’s head, these two streams of information would allow for the building and updating of a 3D egocentric representation of the user’s environment which could subsequently be converted into its corresponding soundscape. For simulated echolocation, the dense distances provided by the depth maps are synonymous with the distances the projected particles travel. In a similar way, these distances could be used to modulate the volume/pulse frequency of the humming sounds. Central to this process will be the *selection* of or emphasis on objects which are relevant to mobility so as not to overwhelm the user with irrelevant audio cues.

Extending this, recent years have seen the proliferation of methods for the fusion of global 3D maps of environments [65, 66, 105–107]. These methods allow for 3D maps of whole environments to be built and updated on-the-fly as the user moves within them. Access to such a map would offer the ability to integrate global-level information of an environment (which may not be available from the user’s local camera view) into the sonification methods—for example, sonifying objects behind the user, objects that dynamically move in and out of the user’s field of view, or objects at far distances or in occluded positions.

Implementing these real-world sonification methods into a wearable SSD presents several challenges. The system must be able to capture, build a representation of the user’s environment, and sonify the relevant parts of the scene all in near real-time. Furthermore, the device must be light in both weight and power consumption, placing significant limits on the computer vision algorithms running on-board the platform. Real-time object detection and recognition methods already exist [104], however, and we can expect increasingly reliable and light-weight solutions to follow in the near future following the rise of household robotic systems and autonomous vehicles.

A crucial consideration in building a wearable audio-based SSD for blind and visually impaired people is the fact that the resultant soundscapes must be delivered in a way that does not mask or impede the ambient sounds on which the user may already heavily rely to perceive their surroundings and remain oriented. Bone-conduction headphones, which transmit sounds through vibrating pads placed on the jaw bones, have been shown to allow equivalent performance in the spatial localisation of audio cues when compared with standard stereo headphones [108], and have been successfully used for auditory spatial navigation through VR environments [74]. This suggests that they do have the potential to accurately represent 3D spatial audio for navigation within a real-world visual-to-audio SSD.

Finally, it is worth noting that another successful real-life implementation of the sonification algorithms presented in this study may yet be entirely virtual. Since the advent of VR environments, an important question has been how to make such environments accessible to the blind and visually impaired community [109]. The focus of VR system development has often been toward a better *visual* experience, but there have been notable exceptions focusing on the non-sighted population [84, 110, 111]. Our auditory VR navigation paradigm can add to the available methods for making virtual worlds more accessible to those without sight.

## 5 Conclusion

The current study has explored the feasibility of two novel visual-to-audio mappings for the task of spatial navigation: simulated echolocation and distance-dependent volume modulation of hums. Both sonification methods were implemented and tested in two virtual reality environments using a head-mounted 3D motion-tracking device. The device created an immersive virtual world in which participants were able to physically walk around virtual scenes. To our knowledge, this is the first work to make use of such an experimental paradigm for the task of spatial navigation, and we believe this approach will be of interest to others working in the dynamics of mobility and spatial navigation. Our key findings showed that participants were able to navigate using both of the proposed sonification methods, with task completion time, velocity, number of collisions and other more nuanced navigational behaviours improving over the course of only six trials. Importantly, these improvements were achieved with participants receiving minimal instruction and a very short training period. This bodes well for future scenarios in which participants have more experience/training with the sonification methods. Although audio-only navigational performance clearly remains below that of visual navigation, our findings suggest that the sonification methods generated an awareness of one’s 3D spatial surroundings. This is a promising step in the direction of enabling independent mobility for visually impaired individuals through sensory substitution.
